# Symbiotic effectiveness of inoculation with *Bradyrhizobium* isolates on soybean [*Glycine max* (L.) Merrill] genotypes with different maturities

**DOI:** 10.1186/2193-1801-3-753

**Published:** 2014-12-18

**Authors:** Anteneh Argaw

**Affiliations:** College of Agriculture and Environmental Sciences, School of Natural Resources Management and Environmental Sciences, Haramaya University, P.O.Box 337, Dire Dawa, Ethiopia

**Keywords:** *Bradyrhizobium*, Genotypes, Maturity group, Soybean [*Glycine max* (L.) Merr

## Abstract

The influence of soybean genotypes with different maturity groups on the symbiotic effectiveness of *Bradyrhizobium* spp under high native soil N is not well known. Therefore, the objective of this work was to evaluate the influence of maturity time of soybean genotypes on the symbiotic effectiveness of *Bradyrhizobium* spp. at higher soil N. Three isolates of *Bradyrhizobium* spp. (UK-isolate, TAL-379 isolate and local-isolate) and six soybean genotypes, three late maturing (Wogayen, TGx-1336424 and Belsa) and three medium maturing (GIZA, Afgat and Gishame) were used for greenhouse experiment. Only GIZA and TGx-1336424 were selected for field experiment. The result of the experiments showed that significantly (P < 0.05) differences in all investigated traits, except total plant tissue N, was observed in TGx-1336424 with UK-Isolate and Local-Isolate.TAL-379 inoculation performed better in all investigated traits of GIZA genotype than other inoculation treatments. N-fertilization in the greenhouse experiment significantly (P < 0.05) improved the shoot biomass of Wogayen and Belsa-95, but did not observe in GIZA and Gishame. The regression analysis obtained between nodule number and nodule dry weight with that of grain yield indicated generally higher R^2^ value for late maturing than that of the medium maturing genotypes. This indicates high importance of nodulation for improving the GY of late maturing genotypes. Hence, this study proves the need for inoculation to improve the production and productivity of soybean sustainably in Ethiopia, with particularly pronounced effect on late maturing genotypes of soybean.

## Introduction

The Leguminosae is one of the most important and largest plant families and is composed of about 750 genera containing 16,000–19,000 species distributed worldwide. Leguminosae has major impacts on agriculture, environment, animal/human nutrition, and health, of which soybean [*Glycine max* (L.) Merr.] is one of the world’s most important and miraculous pulse crops. It accounts for 29.7% of the world’s processed vegetable oil and is rich in dietary protein both for human food and animal feed (Graham and Vance 2003).

Soybean is grown on 10^4^ million hectares of land on five continents with annual total production of 241 million tons and productivity of 2.0 ton ha^-1^ (FAO 2012). In Ethiopia, the area allocated for soybean and the corresponding total annual production has been 31,876 ha and 63,653 tons, respectively, with the productivity less than 2 ton ha^-1^ (CSA 2013), while the potential soybean yield has been estimated to be in the range of 6 – 8 tons ha^-1^ in USA (Specht et al. 1999; Cooper 2003).

Due to its structural requirement for seed protein content, soybean has a high nitrogen demand, often exceeding 200 kg ha^-1^ (Bhangoo and Albritton 1976; Bezdicek et al. 1978; Patterson and LaRue 1983). Biological nitrogen fixation (BNF) and mineral soil or N fertilizers are the main sources for meeting the N requirement of high-yielding soybean varieties. BNF is an effective and efficient source of N supply to plants under favorable atmospheric and environmental conditions (Hungria and Vargas 2000; Chen et al. 2002). More than 50–83% of the necessary N requirement for soybean can be derived from BNF (Salvagiotti et al. 2008; Schipanski et al. 2010) by symbiotic association with either the genus *Bradyrhizobium* or *Sinorhizobium*. Unkovich and Pate (2000) found that, on average, nitrogen fixation by dry-land soybean supplies from 100–142 kg N ha^-1^ on a worldwide basis. Under favourable conditions, levels of nitrogen fixation are elevated more than 250 kg N ha^-1^ (Peoples et al. 1995).

Several research findings clearly showed that soil nitrate repressed nodulation and the effect was magnified as soil nitrate concentrations increased (Mendes et al. 2003; Hungria et al. 2006). However, George et al. (1988) revealed that maturity group of soybean affected the grain yield, soil N uptake and N_2_ fixation. They found that total N and yield of soybean increased with maturity time, which implies that higher total N and yield are obtained from late maturing soybean due to extended vegetative phase. Similarly, Ogoke et al. (2006) have reported increases in N derived from the atmosphere with increasing days to physiological maturity of soybean.

New genotypes of soybean imported from West Africa, USA and Japan were evaluated to identify genotypes well adapted to Ethiopia and, at the same time, to identify potential areas for producing the crop (Amare 1987). During the four decades of breeding effort in the country, genotypes suitable for different areas of production in the country were identified. Soybean breeding in the country, however, has mainly focused on seed yield, disease and shattering resistance, and crop maturity for different areas of production (Asrat et al. 2006). The development of soybean cultivars, which are capable of near-maximum levels of N_2_ fixation in high-NO_3_ soils, remains a high priority in N_2_ fixation research.

In the present investigation, therefore, the influences of soybean maturities group and effectiveness of *Bradyrhizobium* spp., in soil with high N and having no rhizobial association with soybean were thoroughly examined under greenhouse and field conditions using drip irrigation. This research work hypothesized that high soil N decreases the effectiveness of *Bradyrhizobium* sp. inoculation in medium maturing soybean genotypes but may not be observed with late maturing soybean genotypes. Therefore, the specific objective of this piece of research work was to evaluate the influence of soybean genotypes having different maturity time on the symbiotic effectiveness of isolates of *Bradyrhizobium* sp. at high soil N content.

## Materials and methods

### Description of experimental site

The field experiment was conducted in 2012 under irrigation condition at Shinille Agricultural Demostration site, Somali region of Ethiopia, which is semi-arid in nature. The experimental field is located at 09°41′ N latitude and 41°51′ E longitude with an elevation of 1079 meter above sea level. The soils are dominated by sandy clay and increased amount of clay depth by depths. The soils had no history of inoculation with bradyrhizobial strains and soybean cultivation. Previously, the fields were used for maize (*Zea mays* L.) and tomato (*Lycopersicon esculentum* Mill.) production with no history of fertilizer application. There was also no history of any fertiliser application on this site.

Rhizobial population sizes were estimated with the most probable number (MPN) method (Vincent 1970) within 2 weeks of soil sampling, using a base dilution of 10 and the soybean variety Solitaire was used as the trap host. The soil physical and chemical properties and the rhizobial population nodulating soybean were estimated following standard procedures.

### Sources of seeds and inoculum

The soybean genotypes used for this study were provided by the Pawe Agricutural Research Center, Ethiopia, which has been approved to be superior under Ethiopian field conditions. Six soybean genotypes, three of which were late maturing (Wogayen, TGx-1336424 and Belsa) and the remaining three (GIZA, Afgat and Gishame) which were medium maturing, were used for greenhouse experiment. Rhizobial isolates, namely *Bradyrhizobium japonicum* (TAL-379 isolate), *Bradyrhizobium* sp. (UK-Isolate) and *Bradyrhizobium* sp. (local-isolate) were used as inoculants. These isolates were obtained from Holleta Agricultural Research Center (UK- isolate) and National Soil Research Center in Addis Ababa (TAL-379 isolate and local-isolate). The local strain has been previously tested for infectivity under controlled environment at the National Soil Research Center (data not shown).

Sterile fine filter-mud was used as a carrier medium after adjusting the pH to 6.7. The selected isolates of *Bradyrhizobium* sp. were separately incubated in yeast-extract mannitol (YEM) broth at 30°C for 7 days until the concentration reached 10^9^ cells ml^-1^ for inoculants preparation. About 400 ml of *Bradyrhizobium* sp. culture liquid medium was added to 1 kg of the carrier medium, mixed thoroughly, packed in plastic bags. Filter-mud-base inoculum was incubated at 26–28°C for 15 days.

### Pot experiment

Saline soil collected (0–20 cm depth) from an area where the field experiment was carried out at Shinille Agricultural Experimental Site was used for conducting the greenhouse experiment. A plant growth medium containing soil from Shinille Agricultural Experimental Site was prepared based the absence of indigenous bradyrhizobia nodulating soybean to facilitate the visual identification of N-deficiency symptoms in plants nodulated by the specific strain. The soil was collected and dried under aseptic conditions and no rhizobia were detected by a plant infection technique at sowing (Brockwell 1963). Nitrogen (i.e. 20 kg ha^-1^ based) in the form of urea was applied for N treated pots. Six soybean genotypes, three of which were late maturing (Wogayen, TGx-1336424 and Belsa) and the remaining three (GIZA, Afgat and Gishame) were medium maturing, were used for greenhouse experiment.

The pot experiment was conducted in the semi-controlled greenhouse at Haramaya University, eastern Ethiopia, in 2012. Soybean seeds were surface-sterilized with ethanol (1 min) and sodium hypochlorite (NaOCl) (5 min) and then washed several times with deionized water (DW) and five seeds were planted per pot. One week after emergence, each pot was thinned to three seedlings. Pots were regularly watered to 70% water-holding capacity (WHC), while also avoiding water-logging. Rhizobia were cultured to exponential phase in YEM broth, and then 1 ml of culture containing 1 × 10^8^ rhizobial cells per milliliter, was applied to 7-day-old seedlings. The treatment were (i) Local-Isolate, (ii) UK-Isolate, (iii) TAL-379, (iv) N-fertillized pot (pots fertilized with 20 kg ha^-1^ N based), and (v) negative controls (unfertilized and un-inoculated pots), with three replications. The experiment was laid out in completely randomized design (CRD). Plants were harvested 8 weeks after growth at late flowering and early pod setting stage. Harvesting was performed by removing the plants from the pots; the roots were thoroughly rinsed with water, blotted dry on filter paper, and nodules were picked and counted. Total plant and nodule dry weights were recorded after drying at 70°C for 48 hrs.

### Field experiment

The field experiment was conducted at Shinille Agricultural Demonstration Site using well-structured drip irrigation system, in 2012. The land was prepared by deep ploughing, harrowing and levelling. Then the area was ridged and divided into 3 m × 3 m plot. The experimental design was a split plot in a randomized complete block design (RCBD) with three replications. Main plot treatments consisted of four inoculations: UK-isolate, local-isolate, TAL-379 isolate and un-inoculated treatment. Subplot treatments were two soybean genotypes (namely Giza and TGx-1336424). Each soybean cultivar was planted with a spacing of 75 cm between rows, 10 cm between plants, 1.5 m between sub-plots and 2 m between main plots.

Before planting, 20 g of the different bradyrhizobia inoculants was added to each of the different polyethylene bags containing 200 g of soybean seeds. Sugar solution (48%) was added to each bag to enhance proper dispersion, mixing and adhesion of the *Bradyrhizobium* carrier material to the soybean seeds. Two seeds were sown per hill. Plots were immediately irrigated after sowing to ensure moisture and uniform germination. Subsequently, plots were irrigated by a drip irrigation system at 7-day-interval. Weeds were controlled over the growth period with hand hoeing. A set of five plants from each plot was randomly selected at late flowering and early pod setting stage for estimating the nodulation potential (number of nodules and dry weight of nodules) and shoot characteristics (shoot height and shoot dry weight). Dried shoot parts were ground and analyzed for total N using Kjeldahl digestion method.

At physiological maturity, plants were harvested from a 3 m × 2.25 m net plot, leaving two guard or border rows. The plant tops (stalks plus pods) were weighed to determine total dry matter yield before threshing and winnowing to separate the grain, which was then weighed to determine grain yield (GY). Leaves were not included in total dry matter yield determinations, as they had already fallen to the ground. Seed moisture was corrected to 11% when determining grain yield.

### Statistical data analysis

Each sample was analyzed in triplicate and the figures were then averaged. Data were assessed by analysis of variance (ANOVA) using the SAS computer software package ver. 9.1. The least significant difference (LSD) was used to separate treatment means at 5% level of significance. Computer Microsoft Excel Software was used for bar graph and regression analyses.

## Results and discussion

The selected physical and chemical properties of the experimental soil are shown in Table 1. The reaction of plowed soil layer was slightly alkaline with pH value of 7.74. According to Rhoad et al. (1992), the experimental soil could be categorized as saline soil. The EC (4.12 mS/cm) of the soils was very high. The texture of the soil was loamy with higher soil organic matter (SOM), available phosphorus, total nitrogen and investigated cation nutrients.

**Table 1:** **Soil physico-chemical analyses of the field experimental site before sowing at Shinille, Somali Region, in 2012**

Soil properties	Shinille soil
pH in H_2_O	7.74
EC (mS/cm)	4.12
Organic carbon (%)	2.15
Total nitrogen (%)	0.29
Available P (mg kg^-1^)	25.85
Ca (cmol(+) kg^-1^)	31.10
Mg (cmol(+) kg^-1^)	3.22
Na (cmol(+) kg^-1^)	0.14
K (cmol(+) kg^-1^)	2.22
CEC (cmol(+) kg^-1^)	25.90
Zn (mg kg^-1^)	1.19
Fe (mg kg^-1^)	2.2
B (mg kg^-1^)	0.86
NH_4_-N (mg kg^-1^)	26.22
NO_3_-N (mg kg^-1^)	23
Clay (g kg^-1^)	27
Silt (g kg^-1^)	50
Sand (g kg^-1^)	23
Textural class	Loam
Number of rhizobia nodulating soybean	None

Even though the promiscuous soybean genotype (namely TGx-1336424) capable of forming nodule with cowpea rhizobia was used in this experiment, MPN experiment indicated nil or none of rhizobia nodulating soybean existed in Shinille soils. Nevertheless, Okereke and Unaegbu (1992) found that promiscuous soybean was well nodulated by native rhizobia in Nigeria soils though it was symbiotically none effective. Similarly, promiscuous soybean formed nodules in all tested soils of Zimbabwe (Mukutiri et al. 2008). The preliminary experiment conducted by our research group demonstrated the presence of rhizobia nodulating cowpea in Ethiopian soils. The absence of nodules in the present experiment, therefore, could be due to the high soil EC that decreases rhizobial population by affecting the survival and distribution of rhizobia in the soil and rhizosphere (Singleton et al. 1982; Craig et al. 1991).

### Nodulation

Inoculation (I) and genotype (G) x inoculation (I) significantly affected the nodule number (NN) and nodule dry weight (NDW) of all tested soybean genotypes in the field (Table 2) and greenhouse conditions (Tables 3 and 4). A similar finding was also reported by Okereke and Unaegbu (1992) who found interaction effect of variety and rhizobial strain on nodule number in soybean. Inoculation with isolate of *Bradyrhizobium* (Local-isolate) in TGx-1336424 genotype resulted in significantly higher (57.3) NN and NDW (0.4666 g) than other inoculation treatments with *Bradyrhizobium* sp. Significantly higher NN and NDW were also produced by GIZA genotype when it was inoculated with local-isolate and UK-isolate , respectively. The highest NN and NDW produced by GIZA genotype were 47.6 and 0.4300 g, respectively. This indicates the highest NN and NDW produced by both soybean genotypes were induced by the same isolate of *Bradyrhizobium*. Weber et al. (1971), however, showed specificity of strains of *Bradyrhizobium japonicum* sp. with some soybean genotypes. The NN and NDW observed in the present experiment were higher than the data previously reported by Melchiorre et al. (2011) who found that most of the tested genotypes produced less than 7 number of nodules. However, the highest number nodules reported in soybean ranged from 107 to 550 in places where the soils harbored indigenous rhizobia (Shutsrirung et al. 2002). Similarly, it was reported that a field experiment conducted in Nigeria resulted in a maximum NN and NDW that corresponded to 234 and 1.177 g, respectively, and that was induced by indigenous rhizobia (Okereke and Unaegbu 1992).

**Table 2:** **Nodulation status and shoot biomass of TGx-1336424 and GIZA genotypes of soybean inoculated elite isolates of exotic and native** ***Bradyrhizobium*** **spp. in field experiment**

Treatment	NN			NDW			SDW		
	TGx-13364	GIZA	Pooled	TGx-1336644	GIZA	Pooled	TGx-1336644	GIZA	Pooled
TAL-379	35.1b	28.7b	31.9c	0.3473b	0.3098b	0.3286b	69.2ab	61.4a	65.3a
UK isolate	42.2b	46.6a	44.4b	0.3943b	0.4300a	0.4122a	74.4a	50.9b	62.7a
Local	57.3a	47.9a	52.6a	0.4666a	0.4088a	0.4377a	59.8b	48.2b	54.0b
Control	0.0c	0.0c	0.0d	0.0000c	0.0000c	0.0000c	43.0c	52.7ab	47.8b
Mean	33.7	30.8	32.2	0.3021	0.2871	0.2946	61.6	53.3	57.5
F value:									
Inoculation(I)	114.39^***^	81.60^***^	189.79^***^	259.47^***^	149.40^***^	379.72^***^	21.11^***^	4.81^**^	16.35^***^
Genotype (G)			2.97^ns^			2.07^ns^			17.43^***^
G x I			3.44^*^			3.98^*^			11.85^***^
LSD	8.70	9.46	6.3	0.0493	0.0622	0.0386	11.50	9.99	7.4
CV (%)	20.23	24.1	22.1	12.8	17.0	14.9	14.6	14.7	14.7
SEM±	6.81	7.41	7.12	0.0386	0.0487	0.0439	9.00	7.82	8.43

NS- non significant; *significant at 0.05; **significant at 0.01; ***significant at 0.001; Nodule number = NN; Nodule dry weight (g/plant) = NDW; Shoot dry weight at late flowing and early pod setting stage (g/plant) = SDW.

Notes. Means in the same column followed by the same letter are not significantly different at the 5% probability level by Tukey’s test.

**Table 3:** **Nodule number of six soybean genotypes inoculated exotic and native isolates of bradyrhizobia under greenhouse condition in pot experiment**

Treatments	NN						
	GIZA	Afgat	Wogayen	TGx-1336424	Gishame	Belsa-95	Pooled
UK-Isolate	36.3a	35.0a	48.7a	63.3a	41.0a	53.3a	46.3a
TAL-379	20.0b	10.0b	9.7c	6.7b	9.3b	9.0c	10.8b
Local	38.7a	40.7a	36.7b	67.3a	39.0a	29.3a	41.9a
+VE control	0.0c	0.0c	0.0c	0.0b	0.0c	0.0c	0.0c
-VE control	0.0c	0.0c	0.0c	0.0b	0.0c	0.0c	0.0c
Mean	19.0	17.1	19.0	27.8	17.9	18.3	19.8
CV(%)	20.9	17.6	19.6	31.2	14.9	33.2	25.8
LSD	10.7	8.1	10.0	23.0	7.1	16.4	4.8
F value	67.22^***^	124.99^***^	108.77^***^	49.22^***^	179.68^***^	42.52^***^	8.42^***^
SEM±	15.733	9.067	13.800	73.400	7.067	37.133	26.033

NS- non significant; *significant at 0.05; **significant at 0.01; ***significant at 0.001; Nodule number = NN.

Notes. Means in the same column followed by the same letter are not significantly different at the 5% probability level by Tukey’s test.

**Table 4:** **Nodule dry weight of six soybean genotypes inoculated exotic and native isolates of bradyrhizobia under greenhouse condition in pot experiment**

Treatments	NDW						
	GIZA	Afgat	Wogayen	TGx-1336424	Gishame	Belsa-95	Pooled
UK-Isolate	0.2600a	0.2743a	0.3470a	0.4523a	0.2693b	0.3613a	0.3274a
TAL-379	0.4390a	0.1187b	0.0833b	0.0567b	0.1403c	0.0927c	0.1551b
Local	0.2923a	0.3123a	0.2833a	0.4467a	0.3637a	0.2260b	0.3207a
+VE control	0.0000b	0.0000c	0.0000c	0.0000b	0.0000d	0.0000c	0.0000c
-VE control	0.0000b	0.0000c	0.0000c	0.0000b	0.0000d	0.0000c	0.0000c
Mean	0.1983	0.1411	0.1427	0.1911	0.1547	0.1360	0.1606
CV(%)	34.4	15.4	21.3	12.9	21.0	27.5	24.3
LSD	0.1833	0.0584	0.0818	0.0665	0.0873	0.1005	0.0365
F value	24.06^***^	138.87^***^	85.69^***^	275.51^***^	74.60^***^	52.46^***^	7.29^***^
SEM±	0.004651	0.000472	0.000926	0.000612	0.001054	0.001368	0.001519

NS- non significant; *significant at 0.05; **significant at 0.01; ***significant at 0.001; Nodule dry weight (g/plant) = NDW.

Notes. Means in the same column followed by the same letter are not significantly different at the 5% probability level by Tukey’s test.

The research data also proved that higher NN and NDW were produced in the late maturing soybean genotypes than in medium maturing genotypes observed in both greenhouse and field experiment. A similar result was also observed previously by Balatti and Pueppke (1992) who found that the frequency of N_2_-fixing nodules is positively correlated with increasing maturity groups of soybean genotypes. On the contrary, Salvucci et al. (2012) reported that the number and dry mass of nodules are unrelated with maturity length of soybean. Hungria et al. (2006) also found that inoculation responded irrespective of maturity groups between early and medium maturing genotypes. Inhibition effect of native soil N in nodule formation of medium maturing genotype (GIZA) in the present experiment could probably be responsible for these differences as reported by Gibson and Harper (1985). These investigators found that the ability to nodulate in the presence of high N varied with different soybean genotypes and different *Bradyrhizobium* inoculation treatments. Herridge and Betts (1988) have also recognized that four out of the 40 genotypes tested produced higher NN and N_2_-fixation in soils with high N content. Late maturing soybean genotypes supplied adequate carbon allocation, which could promote root growth to explore a larger soil volume below ground continuously for long time, compared to other maturity groups (Hodge 2003). This condition could result in increases in root C exudation, which probably increases survival of inoculated bacteria in the rhizosphere, thereby promoting nodulation. Nodulation status of TGx-1336424 and GIZA in the greenhouse conditions was higher than under field experiment conditions by the same genotypes and with the same inoculation as in greenhouse conditions. This might be due probably to higher number of viable *Bradyrhizobium* isolate inoculated in the pot experiment.

### Total plant tissue nitrogen

Inoculation (I) and G x I interaction induced a significant effect on total plant tissue nitrogen (TPTN) in soybean (Table 5). Several studies have revealed a strong influence of plant variety on N fixation (Herridge and Betts 1985; Neuhausen et al. 1988). Bello et al. (1980) also found that soybean genotype differences accounted from 25 to 70% for variability in the amount of nitrogen fixed per hectare. Though inoculation significantly improved the NN and NDW regardless of the genotypes investigated, no significant improvement was observed in the TPTN in TGx-1336424. Shutsrirung et al. (2002) also found that US type soybean genotype yielded high nodulation with moderately low N2 fixation. This result might be due to low nitrogen-fixing activity or high proportion of ineffective nodules occurring in some soybean genotype-Bradyrhizobium sp association.

All inoculations resulted in significantly high TPTN over the control in GIZA. TPTN varied from 3.9022 to 4.0711% in TGx-1336424, whereasTPTN varied from 3.5656 to 4.0311% in GIZA. TGx-1336424 produced higher TPTN than GIZA in each respective treatment. A similar finding was reported by Neuhausen et al. (1988) who found that the late maturing genotypes have been superior over early genotypes in N2 fixation. These differences have been ascribed largely to higher biomass (Bushby and Lawn 1992) and longer duration of growth and development in the field (Eaglesham et al. 1982). The higher TPTN in TGx-1336424 could also be the outcome of higher nodule formation (Herridge and Betts 1988; Abi-Ghanem et al. 2011). This finding indicates and emphasizes the need for provision of higher N either from soil or from symbiotic N2 fixation in late maturing soybean genotypes. Variation among genotypes suggests it would be possible to breed soybean for increased N2-fixation that has a potential to improve soil fertility for sustainable crop production.

### Shoot dry weight at late flowering and early pod setting stage

Of the six investigated soybean genotypes, four genotypes (i.e. Afgat, Belsa-95, Gishame and Wogayen) displayed significant variation in shoot dry weight (SDW) among inoculation treatments (Table 6). Significant effect of inoculation was observed on SDW in TGx-1336424 only in the field experiment (Table 2). SDW of GIZA, however, was not significantly improved by inoculation as was observed in the greenhouse conditions. Nodulation did not also significantly improve in spite of inoculation. A similar finding was also reported by Abi-Ghanem et al. (2011).

**Table 6:** **Shoot dry weight of six soybean genotypes inoculated exotic and native isolates of bradyrhizobia under greenhouse condition in pot experiment**

Treatments	SDW						
	GIZA	Afgat	Wogayen	TGx-1336424	Gishame	Belsa-95	Pooled
UK-Isolate	7.833a	8.233a	9.933a	8.500a	9.133a	7.667a	8.550a
TAL-379	8.767a	7.267a	6.500b	6.033a	6.833ab	7.167ab	7.094b
Local	8.367a	7.733a	9.600a	9.300a	6.300ab	8.100a	8.233a
+VE control	8.600a	8.200a	9.800a	7.900a	6.467ab	8.367a	8.222a
-VE control	6.367a	4.633b	7.100b	6.467a	5.900b	5.367b	5.972c
Mean	7.987	7.213	8.587	7.640	6.927	7.333	7.621
CV(%)	19.8	12.4	6.4	17.2	16.3	10.8	14.5
LSD	4.258	2.4045	1.488	3.532	3.038	2.127	1.031
F value	1.13^ns^	8.38^***^	26.60^***^	3.27^ns^	3.84^*^	6.78^**^	4.31^***^
SEM±	2.5107	0.8007	0.3067	1.7273	1.2780	0.6267	1.2083

NS- non significant; *significant at 0.05; **significant at 0.01; ***significant at 0.001; shoot dry weight at late flowering stage and early pod setting stage (g/plant) = SDW.

Notes. Means in the same column followed by the same letter are not significantly different at the 5% probability level by Tukey’s test.

High nodulation in high soil N condition may not have direct advantage on yield improvement (Song et al. 1995). However, the late maturing genotype (TGx-1336424) produced the highest SDW (74.4 g). This is consistent with research finding by Papakosta and Veresoglou (1989) who reported that dry matter accumulation of soybean cultivars was influenced by maturity groups. It is also obvious that nodulation has a good agreement and positive correlation with SDW in late maturing genotypes (Sogut 2006). Furthermore, the non-nodulating mutant of late maturing genotypes could produce higher SDW in high soil N condition than other genotypes (Herridge and Betts 1988). N-fertilization did not significantly improve the SDW of medium maturing genotypes (GIZA and Gishame), but showed a significant effect on SDW of late maturing genotypes (Wogayen and Belsa-95) under greenhouse condition, except TGx-1336424. In contrast, Sogut (2006) found that N-fertilization produced inferior biomass yield compared to inoculation treatments irrespective of maturity groups. According to the description and classification by Bruce and Raymont (1982), the soil utilized for these experiments had higher soil total nitrogen, which could be enough for maximum shoot biomass production for medium maturing genotypes. Consequently, further addition of exogenous N might have no further improvement in SDW.

The less effective *Bradyrhizobium* isolate (TAL-379 isolate) did not significantly improve the SDW of two of investigated late maturing genotypes. This illustrates the importance of use of highly effective isolate of *Bradyrhizobium* sp. for significant improvement in late maturing soybean genotypes. Furthermore, there was a non-significant effect of N-fertilization on SDW in TGx-1336424. These results are in agreement with the findings of Herridge and Brockwell (1988) who found that soil N was not always complementary with N_2_-fixation in meeting the N requirements of the growing crop. Non-responsiveness for N-fertilization could be due to insufficient supply of N to the crop at late flowering and early pod setting stage of soybean.

**Figure 1:**
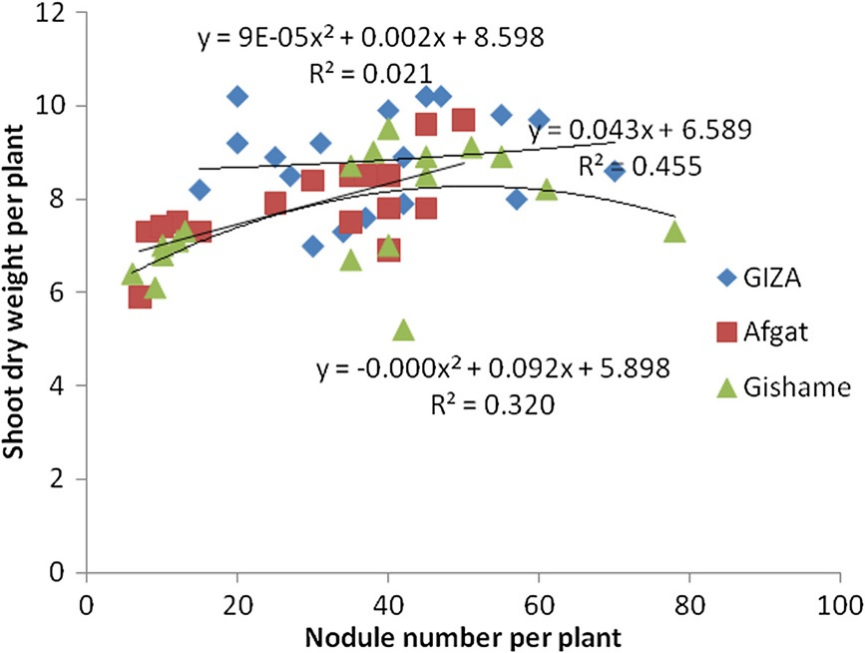
**Regression of shoot biomass yield at late flowering stage of medium maturing genotypes of soybean on nodule number over all inoculation treatments under greenhouse condition.**

**Figure 2:**
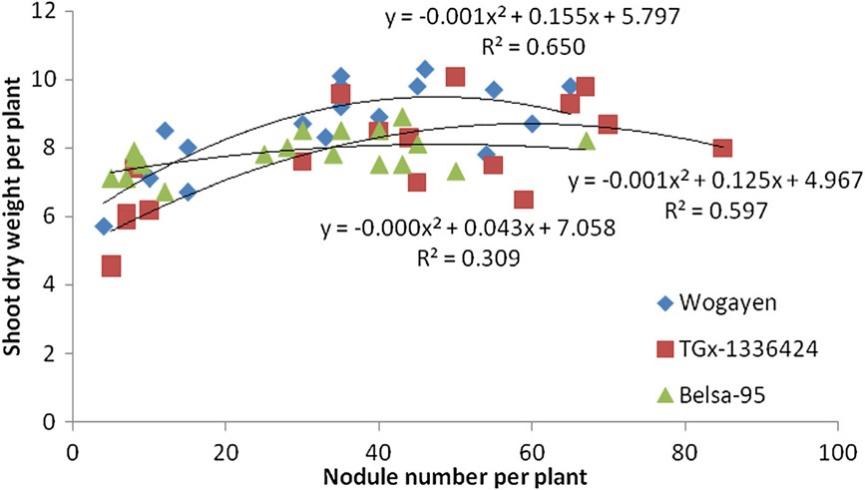
**Regression of shoot biomass yield at late flowering stage of late maturing genotypes of soybean on nodule number over all inoculation treatments under greenhouse condition.**

**Figure 3:**
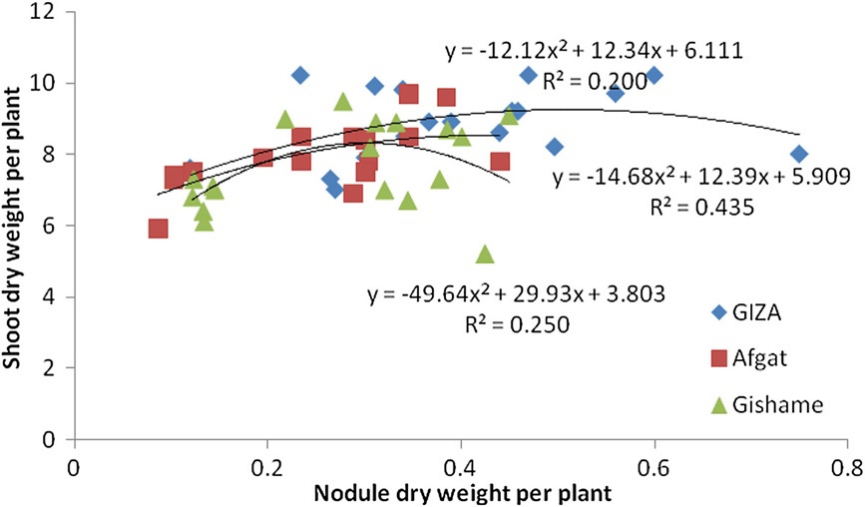
**Regression of shoot biomass yield at late flowering stage of medium maturing genotypes of soybean on nodule dry weight over all inoculation treatments under greenhouse condition.**

**Figure 4:**
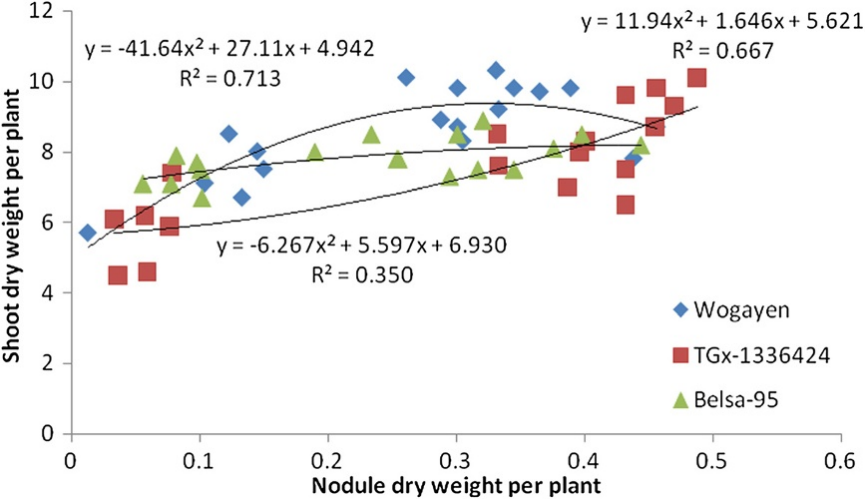
**Regression of shoot biomass yield at late flowering stage of late maturing genotypes of soybean on nodule dry weight over all inoculation treatments under greenhouse condition.**

The research data generated from the greenhouse experiment showed that soybean NN had a significant association with SDW in five out of six soybean genotypes tested (Figures 1 and 2). The highest coefficient of determination was also obtained from late maturing genotypes under greenhouse conditions. The late maturing Wogayen exhibited the highest R^2^-value (R^2^ = 0.650), followed by TGx-1336424 (R^2^ = 0.597). This indicates the importance of induced nodules in late maturing soybean genotypes. It is obvious that nodulation has a positive correlation with N_2_-fixation, production and productivity of leguminous plants (Okereke et al. 2001). The medium maturing soybean genotype (GIZA), however, displayed a non-significant association between SDW and NN. Furthermore, all tested soybean genotypes had a significant and quadratic relationship between SDW and NDW under greenhouse conditions (Figures 3 and 4). The highest coefficient of determination was R^2^ = 0.667 exhibited by late maturing genotypes namely Wogayen genotype followed by TGx-1336424 (R^2^ = 0.713). The medium maturing soybean genotypes, namely GIZA and Gishame, had the lowest coefficients of determination (R^2^) of 0.2000 and 0.250, respectively. The higher R^2^-value between SDW and NDW indicates that the mean variation of SDW was mainly determined by the NDW rather than the NN of late maturing genotypes. It is known that high NDW is associated with high production and productivity of soybean (Okogun et al. 2005). In contrast, Pulver et al. (1982) showed that inoculation of the promiscuous soybean varieties increased nodule mass but did not significantly increase shoot dry weight at 60 days after planting.

### Plant height, number of pods per plant and number of seeds per pod

Inoculations had significant (p ≤ 0.05) effect on plant height at late flowering and early pod setting stage (PH_1_) and at harvest (PH_2_) (Table 7). A similar finding was reported by Egamberdiyeva et al. (2004) who found that all investigated genotypes of soybean resulted in significant improvement in plant height by inoculation with *Bradyrhizobium* sp. Moreover, genotype x inoculation interaction was also significant in PH_1_ but not in PH_2._ This indicates that plant height at late flowering stage was affected by maturity groups of soybean genotypes and inoculated isolate type of *Bradyrhizobium* sp. Though the TAL-379 isolate induced inferior nodules, all inoculations with TAL-379 isolates produced significantly higher PH_1_ in all investigated soybean genotypes than in the control. But at the later stage, inoculation with the most effective isolate (local-isolate) produced significantly higher PH_2_ in the late maturing genotype (TGx-1336424) than in the control, but all inoculations, including inoculation with TAL-379 isolate, resulted in statistically equal PH_2_ in medium maturing genotypes. This indicates that very effective symbiotic association between *Bradyrhizobium* sp. and late maturing genotypes is important for significant improvement of pertinent traits at harvest. Okereke et al. (2001) indicated that productivity of soybean could be determined by the interaction between the soybean and the rhizobia strains.

**Table 7:** **Yield components of TGx-1336644 and GIZA genotypes of soybean inoculated elite isolates of exotic and native** ***Bradyrhizobium*** **spp. in the field experiment**

Treatment	PH _1_			NPP			NSP			PH _2_		
	TGx-1336644	GIZA	Pooled	TGx-1336644	GIZA	Pooled	TGx-1336644	GIZA	Pooled	TGx-1336644	GIZA	Pooled
TAL-379	70.7a	71.3a	71.0a	134.7a	176.1a	155.4a	2.626a	2.700a	2.663a	77.1ab	81.7a	79.4a
UK Isolate	73.0a	61.0ab	67.0ab	145.9a	153.3b	149.6a	2.624a	2.662ab	2.643a	82.1ab	80.3a	81.2a
Local	61.8b	56.9b	59.3c	149.8a	153.0b	151.4a	2.367ab	2.692a	2.529a	86.4a	75.0a	80.7a
Control	61.2b	64.0ab	62.6bc	115.0b	102.9c	108.9b	2.180b	2.367b	2.273b	69.7b	64.8b	67.2b
Mean	66.7	63.3	65.0	136.3	146.3	141.3	2.449	2.605		78.8	75.4	77.1
F value:												
Inoculation (I)	7.34^**^	4.71^**^	8.06^***^	10.09^***^	29.54^**^	33.45^***^	8.28^**^	3.95^*^	10.56^***^	2.77^ns^	8.82^**^	6.98^**^
Genotype (G)			3.51^ns^			7.06^*^			8.02^**^			1.81^ns^
G x I			3.39^*^			9.01^***^			1.38^ns^			1.74^ns^
LSD	8.55	10.8	6.69	18.8	21.8	14.0	0.2888	0.3084	0.206	16.58	9.9	9.40
CV (%)	10.05	13.3	11.7	10.8	11.7	11.3	9.23	9.3	9.3	16.5	10.3	13.9
SEM±	6.70	8.43	7.61	14.7	17.07	15.95	0.2261	0.2414	0.2339	12.97	7.74	10.7

NS- non significant; *significant at 0.05; **significant at 0.01; ***significant at 0.001; Plant height at late floweing and early pod setting stage (cm) = PH_1_; Number of pods per plant = NPP; Number of seeds per pod = NSP; Plant height at harvest (cm) = PH_2._

Notes. Means in the same column followed by the same letter are not significantly different at the 5% probability level by Tukey’s test.

Number of pods per plant (NPP) significantly varied with inoculation (I), genotypes (G), and I x G interaction (Table 7). Inoculation x genotype interaction indicates that responses of soybean genotypes differ in NPP with different inoculation treatments. NPP varied from 115 in the control plots to 149.8 in plots inoculated with local-isolate on TGx-1336424. The NPP varied from 102.9 in the control to 176.1 in GIZA inoculated with TAL-379 isolate. Similarly, Patra et al. (2012) found that inoculation with *Bradyrhizobium* sp. significantly improved the NPP in soybean. The investigators also indicated that inoculation improved NPP by 45.8% over the control. Sarafi (1978) also reported that number of pods per plant has often been consistently and positively correlated with productivity of bean. Significant effect of inoculation and genotype was also observed in the number of seeds per pod.

Generally, inoculation with isolates of *Bradyrhizobium* sp. resulted in a significant increase in the NSP in both genotypes over the control. NSP varied from 2.626 inoculated with TAL-379 isolate to 2.180 in the control in the TGx-1336424. The NSP in GIZA ranged from 2.700 inoculated with TAL-379 isolate to 2.367 in the control pots. A similar finding was also reported in Ethiopia by Tamiru et al. (2012) who found that inoculation with TAL-379 isolate significantly improved the number of seeds per pod in soybean. On the contrary, it has been reported that NSP of soybean has not been significantly improved by inoculation with *Bradyrhizobium* sp (Patra et al. 2012).

### Total biomass yield

Total biomass yield (kg/ha) (TBY) was significantly influenced by inoculation (I), genotype (G) and I x G interaction (Table 7), thereby indicating the importance of selection and inoculation with appropriate bradyrhizobia strains for different soybean genotypes. Inoculation with UK-isolate and local-isolate in TGx-1336424 resulted in significantly higher TBY than other treatments. Although inoculation with TAL-379 isolate performed poorly in nodulation, inoculation of GIZA with the same isolate produced significantly higher TBY than in the control, thereby indicating the specific association of the soybean genotypes with *Bradyrhizobium* strains. The host genotype-microsymbiot interaction has influence on the shoot biomass productivity of soybean (Okereke et al. 2001).

**Table 5:** **Yield and plant total tissue nitrogen of TGx-1336644 and GIZA genotypes of soybean inoculated elite isolates of exotic and native Bradyrhizobia spp. in field experiment**

Treatment	TBY			GY			TPTN		
	TGx-1336644	GIZA	Pooled	TGx-1336644	GIZA	Pooled	TGx-1336644	GIZA	Pooled
TAL-379	6604.9b	8374.5a	7489.7a	2259.14b	1503.89c	1881.51c	4.0267a	4.0311a	4.0289a
UK Isolate	9207.8a	6913.6b	8060.7a	2518.40a	2205.90a	2766.40a	4.0711a	4.0244a	4.1578a
Local	9032.9a	6049.4bc	7541.2a	2519.18a	2277.33a	2398.25b	4.0544a	4.0200a	4.0372a
Control	5535.0c	5411.5c	5473.3b	1722.39c	1319.05b	1520.72d	3.9022a	3.5656b	3.7339b
Mean	7593.2	6687.2	7141.2	2254.77	1826.53	2141.72	4.0136	3.9654	3.9890
F value									
Inoculation (I)	43.68^***^	17.05^***^	30.32^***^	68.10***	142.26^***^	191.96^***^	1.28^ns^	8.52^**^	9.18^***^
Genotype (G)			19.18^***^			32.37^***^			0.66^ns^
G x I			27.18^***^			43.96^***^			3.18^*^
LSD	1052.8	1189.7	773.3	174.32	288.60	148.2	0.2593	0.3751	0.222
CV (%)	10.9	13.9	12.3	6.05	12.37	7.9	5.06	7.4	6.3
SEM±	824.3	931.3	879.5	136.48	225.97	168.6	0.2030	0.2937	0.2524

NS- non significant; *significant at 0.05; **significant at 0.01; ***significant at 0.001; Total biomass yield (kg/ha) = TBY; Grain yield (kg/ha) = GY; Total plant tissue N(%) = TPTN.

Notes. Means in the same column followed by the same letter are not significantly different at the 5% probability level by Tukey’s test.

The TBY of inoculated TGx-1336424 varied from 9207.8 kg ha^-1^ to 5535.0 kg ha^-1^ in the control, with an average value of 7593.2 kg -ha^-1^. The highest TBY of this genotype was higher by 66.4% than the value in the control. The value of TBY also varied from 8374.5 kg ha^-1^ in the inoculated GIZA to 5411.5 kg ha^-1^ in control, with average value of 6687.2 kg ha^-1^. The highest TBY in GIZA was 54.8% over the control of the same genotype. This in general indicated higher improvement of TBY in inoculated late maturing genotypes than the medium maturing genotypes. Similarly, Sogut (2006) found that inoculation with *Bradyrhizobium* sp. has been more effective in late maturing genotypes than in medium and early maturing genotypes.

**Figure 5:**
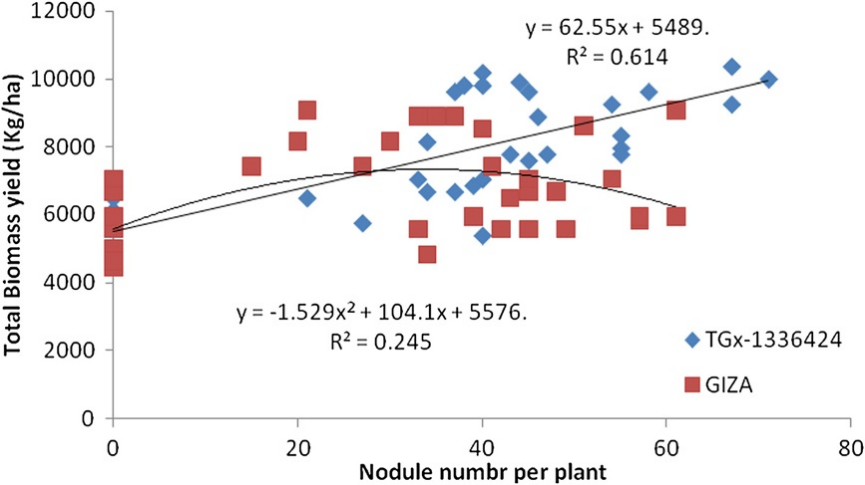
**Regression of total biomass of soybean genotypes, GIZO and TGx-1336424 on nodule number over all inoculation treatments under field condition in Shinille, Somali region, Ethiopia.**

**Figure 6:**
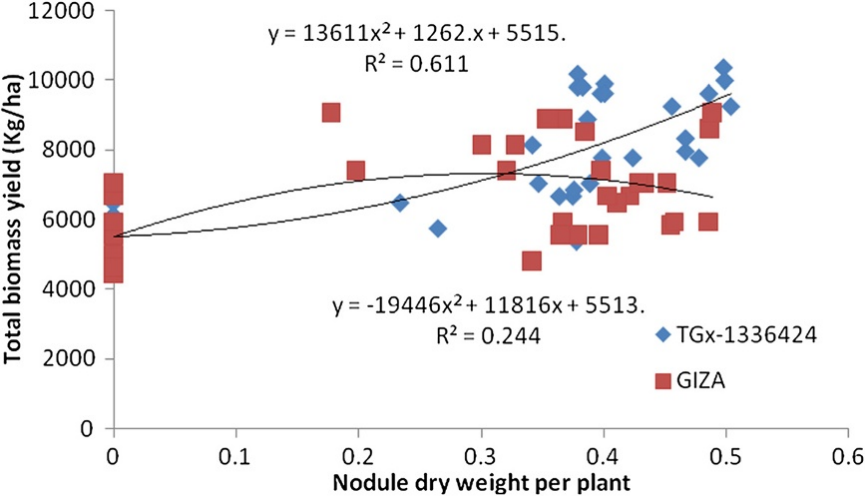
**Regression of total biomass yield of soybean genotypes, GIZO and TGx-1336424 soybean genotypes on nodule dry weight over all inoculation treatments under field condition in Shinille, Somali region, Ethiopia.**

It was found that NN and NDW had a significant association with TBY (Figures 5 and 6). Significant (p ≤ 0.05) linear (Y = 62.55x + 5489) and quadratic (Y = -1.529x^2^ + 104.1x + 5576) associations were observed between NN and TBY in TGx-1336424 and GIZA genotypes, respectively, indicating that linear increases of NN could lead to further linear increases of TBY in TGx-1336424. Shutsrirung et al. (2002) found that the symbiotic efficiency depended largely on the soybean cultivars. The genotype TGx-1336424, however, displayed higher coefficient of determination (R^2^ = 0.614), followed by GIZA genotype (R^2^ = 0.245). NDW also had a significant association with TBY, displaying quadratic relationships, such as Y = 13611x^2^ + 1262x + 5515 and Y = -19446x^2^ + 11816x + 5513 for TGx-1336424 and GIZA, respectively. TGx-1336424 had higher coefficient of determination (R^2^ = 0.611), followed by GIZA (R^2^ = 0.244) as detected with regression analysis between TBY and nodulation.

### Grain yield

Inoculations (I), genotypes (G) and I x G interaction significantly (p ≤ 0.05) influenced grain yield (GY) in soybean (Table 7). Inoculation with isolates of *Bradyrhizobium* sp. resulted in a significant improvement of GY of both genotypes over the control. The GY of TGx-1336424 varied from 1722.39 to 2519.18 kg ha^-1^ due to inoculation with local-isolate, with an average value of 2254.77 kg ha^-1^. GY of GIZA also varied from 1319.05 to 2277.33 kg ha^-1^ due to inoculation with UK-isolate, with an average value of 2028.67 kg ha^-1^. The separate GY of both genotypes obtained from inoculation was higher than the GY of the same genotypes managed with recommended N fertilizer (Asfaw et al. 2006). The investigators found 1735.1 kg ha^-1^ GY for TGx-1336424. Similarly, Shrivastava et al. (2000) reported the beneficial effect of seed inoculation with *Rhizobium* sp. and enhanced soybean yield by an average of 11% over the recommended N fertilizer.

The maximum GY in TGx-1336424 and GIZA was higher by 46.3 and 72%, respectively, than the GY of the control treatment. However, the data indicated that the highest GY of TGx-1336424 had 241.85 kg ha^-1^ yield advantage over the highest GY of GIZA. The yield increase due to inoculation could be attributed to the long-lasting N fixation and N uptake by soybean plants. Sogut (2006) found that late maturing genotypes generally resulted in higher grain productivity than the other maturity groups. Seed yield is also highly dependent on the length of development, particularly on the duration of the pod filling phase (Gay et al. 1980; Smith and Nelson 1986). This might be due to the formation of new nodules and their development at later growth stages, i.e. the pod-filling stage (Shutsrirung et al. 2002). Based on the present findings, it can be concluded that despite the availability of adequate soil N, symbiotic N does, indeed, increase yield in late maturing soybean genotypes.

**Figure 7:**
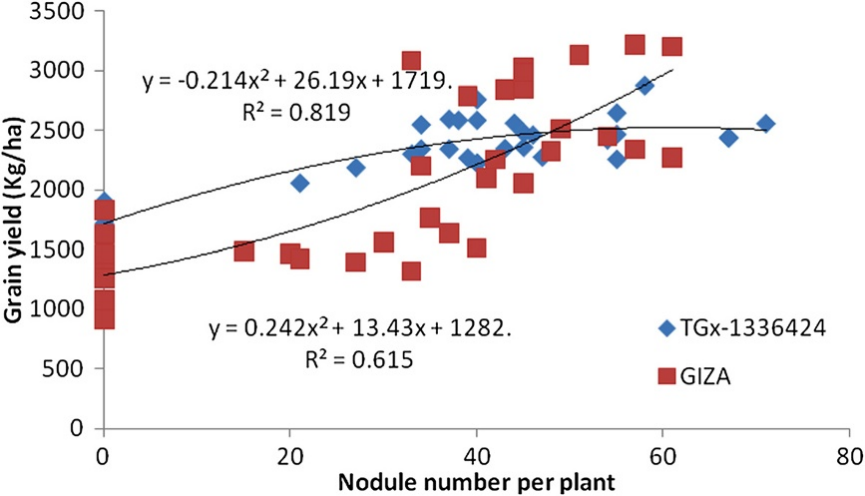
**Regression of grain yield of soybean genotypes, GIZO and TGx-1336424 soybean genotypes on nodule number over all inoculation treatments under field condition in Shinille, Somali region, Ethiopia.**

**Figure 8:**
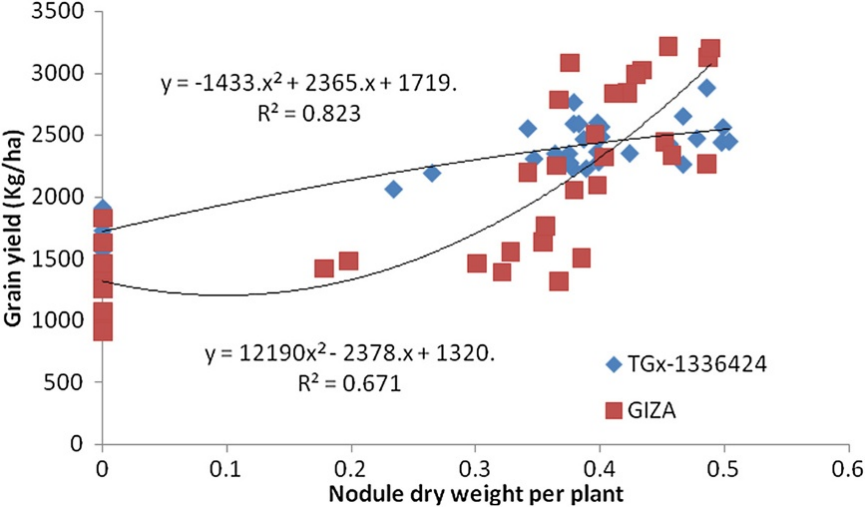
**Regression of grain yield of soybean genotypes, GIZO and TGx-1336424 soybean genotypes on nodule dry weight over all inoculation treatments under field condition in Shinille, Somali region, Ethiopia.**

The NN and NDW of both soybean genotypes had a significant association with GY (Figures 7 and 8). The GY of both genotypes had a quadratic association with increasing NN and NDW. TGx-1336424 had a higher coefficient of determination with NN (R^2^ = 0.819) and NDW (R^2^ = 0.823), indicating the importance of nodulation for grain yield increase in late maturing genotypes. NN and NDW of TGx-1336424 accounted 81.9 and 82.3%, respectively, for variation in GY. This indicates the importance of nodulation for GY increase in late maturing soybean genotypes than in the medium maturing genotypes.

## Conclusions

The findings of experiments conducted under greenhouse and field conditions clearly indicated the significant effects of genotype, inoculation and genotype x inoculation interaction on nodulation and other investigated soybean parameters. The data also indicated significant differences in the performance of isolates of *Bradyrhizbium* spp. in different soybean genotypes with different maturities. The regression analyses indicated the existence of significant association between nodulation and GY manifested with higher R^2^-values in late maturing soybean genotypes than in the medium maturing genotypes. This indicates the need for consideration of maturity times beside soybean genotypes for the selection of effective and efficient isolates of *Bradyrhizobium* spp. The results also indicate pronounced effects of inoculation with isolates of *Bradyrhizobium* spp. on late maturing genotypes though the soil had higher total N. Further research works to soil fertility problem in relation to saline soil would be suggested to improve the productivity of soybean by reducing the yield gap.
